# Is consumer neural response to visual merchandising types different depending on their fashion involvement?

**DOI:** 10.1371/journal.pone.0241578

**Published:** 2020-12-23

**Authors:** Hyoung-Sukh Kim, Jin-Hwa Lee, So-Hyeon Yoo

**Affiliations:** 1 College of Design, Dongseo University, Busan, Korea; 2 Department of Clothing & Textiles, College of Human Ecology, Pusan National University, Busan, Korea; 3 School of Mechanical Engineering, Pusan National University, Busan, Korea; Tohoku University, JAPAN

## Abstract

This study investigated consumers' responses to fashion visual merchandising (VM) from a neuroscientific perspective. The brain activations of 20 subjects differently involved in fashion were recorded using functional near-infrared spectroscopy in response to three different fashion VM types. According to the types of fashion VM, significant differences were observed, which were significantly higher for the creative VM. Moreover, highly fashion-involved subjects showed activation of the orbital frontal cortex region in response to the creative VM. Based on these results, it is suggested that marketing strategies should be devised explicitly for the brand's targeted audience and goals.

## Introduction

The role of off-line stores has been rapidly changing in an omnichannel environment, and companies are striving to capture consumers' imagination and provide memorable experiences. In the fashion industry, off-line stores are the place where companies communicate directly with consumers, and these stores are represented through visual merchandising (VM). Currently, many fashion companies are actively using VM as a strategic marketing approach to establish the in-store image of a brand by differentiating their corporate image and raising product preference [1,2]. Consumers are more likely to make an impulsive purchase decision when exposed to visual stimuli [3]. Ebster and Garaus [4] defined VM as an art and science of displaying products in the most visually appealing way and insisted that a focus should be placed on communication with consumers through images and presentations. When it comes to fashion VM, it is crucial to elaborate on a scientific and strategic approach, which should be appropriate given the target and purpose. To plan an effective VM strategy for fashion companies, it is necessary to understand the various consumers' minds objectively for each VM type, based on their understanding of the VM type. Most human behaviors, if not all, either begins as an unconscious process or occurs entirely outside of conscious awareness [5]. Also, the human mind should focus on producing services or products that are more valuable to humans by better understanding how it is unconscious and implicit than the ones in the outside world. Aware of such unconscious consumer responses, neuroscience is now being applied to marketing in numerous areas. There is also an approach in fashion using brain science Touchette & Lee, 2017; Baldo et al. [6,7], and it has been confirmed that positive(likable) and negative(unlikable) VMs in fashion stores can be distinguished through brain science methods [8]. The neuroscientific approach to fashion VM can provide scientific evidence for a customized store strategy based on consumers' characteristics, as it can identify the emotional aspects of individual consumers and their cognitive behaviors.

Also, consumers' fashion involvement plays a crucial role in making purchasing decisions for fashion products. Influencers and trendsetters often display much higher levels of engagement, and their utility for expanding the brand's reach is crucial. This paper will explore consumers' responses to three fashion VM types: fact-based, corrected-processing, and creative. It will use neuroscience to provide a scientific foundation for the creation of strategically targeted VM.

## Neuroscientific approach in fashion

### Visual merchandising and consumer emotion

Visual merchandising in fashion stores is a useful promotional tool, which enables differentiation from other competitive brands by presenting consumers with visual information and image about the brand and products [9]. VM also provides visualization for integrating brand identity, product policy, planning, and delivery method [10]. Consumer awareness about VM can spark interest and motivate further exploration of products in the market [11]. Appropriate VM can also induce a series of behaviors, such as consumption and store/product recognition [1]. Besides, Besides, Wright et al. [12] reported that consumers' participation with the store could be increased and lead to favorable buying behaviors by creating an atmosphere that satisfies the practical and emotional needs of the consumers. In summary, there is a significant correlation between VM and consumers' purchasing behavior, and VM is an essential factor regarding the brand that it is ultimately purchased [13].

Emotion is another critical element that has been often highlighted in the field of consumer behavior [14]. Emotions focus on an object and are associated with specific behavioral responses [10]. When experienced in the store, feelings are caused by the interaction between the environment and consumers. They can have a unique impact [15], influencing consumers' satisfaction level and other behaviors, such as purchasing [16]. Also, emotions and preferences created due to visual stimuli, including the store environment, are influenced by how well consumers process the information delivered to them [17,18]. In other words, knowledge about the mood of consumers in a marketing context enables marketers to understand them and their response to marketing strategies fully, which can be influenced by marketing tactics such as service encounters, point-of-purchase stimuli, and communications [15].

### Consumer behavior and brain science

Over the last two decades, researchers in the social cognition field have tried to identify the effects of environmental clues on human behavior [19–23]. One such noteworthy theory is the "perception-behavior link," which states that a given perception about the social environment or stimuli can make people behave accordingly [24,25]. This is grounded in the fact that the premotor cortex, which is responsible for perception in the human brain, is closely associated with the area which governs behavior. Due to this link, perception influences behavior directly and unconsciously [24].

With recent advances in brain imaging techniques, neuroscience has developed at a fast pace, accelerating a diverse array of interdisciplinary studies [8]. Neuroscience is being applied to human and social studies, including law, economics, business administration, education, and the natural sciences. These studies seek to uncover the genetic influence and provide an in-depth explanation of the causes and reasons for human behavior.

As neuroscience proves itself more useful and effective than conventional methods in providing information about marketing, it has received more considerable attention from business people as well as researchers. Thanks to this focus, we are now able to understand how the brain functions when consumers experience a marketing stimulus, how it responds to a marketing stimulus in various contexts, and how it interprets this response and influences decision-making and behavior. Such a neuro-scientific approach is especially promising when applied to marketing problems such as looking into the neural circuits related to consumer decision-making or understanding the role of attention [26–29].

One advantage is that this approach enables researchers to look at the response of people during a short time [30]. Recent studies involving the fashion industry include Touchette and Lee [6], who investigated the neural mechanism of the appeal of apparel products, and Kim and Lee [8], who confirmed that positive/negative VM stimuli could be classified as a brain response. Baldo et al. [7] suggested a new approach based on neural data to predict product quality and discuss the importance of market prediction in the retail footwear industry. In summary, neuroscience has proven useful in understanding the hidden feelings of consumers beyond their rational judgment, and it is essential to strategically create VM to fit the needs of different consumers to captivate them and elicit wanted behaviors.

### VM types

The types of fashion VM have been classified variously depending on the industry or research perspectives, and no single objective classification has yet been established. Kwon and Shin [31] categorized product group display and image group display and analyzed attitude factors toward apparel window displays by the gender of consumers. Cornelius et al. [32] delineated two classifications, merchandise-focused display and artistic display, and studied their separate effects on the retail store image. Park and Jeon [33] researched consumers' responses dividing VM into "sensual" and "utilitarian," while Lee [34] shared VM into "utilitarian" and "hedonic" in the article entitled A Study on the Influence on VM based on Consumers' Regulatory Focus on Shopping Value and Shopping Satisfaction. As such, most studies on fashion VM have adopted a classification from dichotomous or aesthetic perspectives.

Storytelling-based classification is also widely used in brand, advertising, and space, among other areas. Oh [35] divided storytelling into the nature of the product, relational information, and myth types, and applied them to study consumers' attitude. Cho [36] categorized advertising into real storytelling, story twisting, and story-making. More recently, Kim and Lee [37] applied storytelling to differentiate the fact-based, corrected-processing, and creative types of VM from a marketing perspective, while Lee [38] conducted research on flexible identity design using the same three models.

Therefore, this study produced stimuli based on the three VM types (fact-based, corrected-processing, and creative types) identified in the recent study and defined differences between the three VM types, as shown in Table 1.

**Table 1:** Definition of 3 VM types.

VM Types	Definition	Method of expression
Fact-based type	The type of Visual Merchandising that is based on actual experience or events.	realistic, symbolic
Corrected-processing type	The type of Visual Merchandising that connects to the object of value (brand or commodity) and the actor and situation surrounding it.	moody, informative
Creative type	The type of Visual Merchandising that makes a story that does not exist on a new story that fits the characteristics of a brand or product	imaginative, surreal

Kim and Lee (2017) [37].

### Consumers’ fashion involvement

Consumers' fashion involvement is defined as the degree of interest or perceived importance in an object or a situation, with high or low involvement affecting the decision-making and evaluation processes of consumers [39–40]. Fashion involvement is a significant antecedent influencing fashion trends and purchasing behavior, and it is also used as a variable to distinguish different groups of fashion product consumers [41]. The recognition of the product, the first step of the decision-making process, is shaped through diverse communication channels such as advertising, store display, or information obtained from other consumers. Highly fashion-involved consumers are quicker to recognize the product and develop interest, and their level of interest is also stronger than that of low-involved consumers [42]. In the fashion industry, where rapid trend changes make it challenging to predict consumers' behaviors, highly fashion-involved consumers should be considered vital for establishing marketing strategies as they are likely to be drivers and influencers [43–44].

## Hypotheses

Cornelius et al. [32] stated that more innovative displays achieve better image valuations and store image benefits from the presence of a storefront display. In addition, Oh and Petrie [45] found that shoppers have different reactions to the two types of window display; the merchandising display supports understating, and the artistic display promotes exploration. However, these studies have mostly been conducted based on a dichotomous classification using questionnaires and, therefore, a new scientific approach is required. Against this backdrop, the following hypotheses have been elaborated:

### Hypothesis 1

There would occur significantly different brain activations (i.e., oxy-hemoglobin concentration levels) when consumers are exposed to different types of VM (fact-based, corrected-processing, and creative fashions).

Highly fashion-involved consumers tend to have more knowledge as well as more interest in fashion stores and products than low-involved consumers [40]. Also, novices without prior experience or relevant training prefer simple stimuli, while experts with ample experience and training prefer complex stimuli [18], which is in line with the findings of Vitz [46] and confirms a repeated exposure enhanced preference for complex stimuli. In summary, a substantial amount of research found that highly involved consumers given specific products or situations pay more attention to and better understand various information to which they are exposed in the shopping environment than low-involved consumers [47–50].

Lim [51] classified the VM types of the Speciality store retailer of Private label Apparel (SPA) brands into "utilitarian" and "emotional" and examined store attitude according to the different VM types: Low-sensation seekers scored higher in-store attitude for utilitarian VM while high-sensation seekers scored higher for emotional VM. Also, high-sensation seekers showed a greater tendency for impulse buying. Therefore, it can be assumed that brain activation will be different depending on the level of fashion involvement.

### Hypothesis 2

Depending on consumers' levels of fashion involvement, the features of the hemodynamic responses in the prefrontal cortex are different upon three VM types.

Higher mental activity occurs mainly in the outer cerebral cortex, which is divided into areas responsible for various functions. Recently, greater attention has been paid to the prefrontal lobe, which is presumed to carry out executive functions related to planning and decision-making. The prefrontal cortex (PFC) is divided into the dorsolateral prefrontal cortex (DPFC) and the orbital frontal cortex (OFC). DPFC is associated with "cold cognition," or thought processes such as perception, short-term memory, executive memory, planning, and regularity.

The critical difference is that the OFC controls emotional processes, whereas the DPFC controls perceptual and memory processes. Thus, the prefrontal cortex as a whole is involved in executive control of information processing in many domains [52–58]. While involved with similar functions as those of DPFC, the OFC is mostly responsible for "hot cognition," such as emotion and empathy. Sul [59] suggested that OFC plays an important role in making decisions based on values and renewing the value of the expected outcome from the chosen behaviors. Also, OFC is a cerebral structure involved in both reward and punishment, and decision making, which [60] associated with positive emotions, and increased oxygen saturation through the use of functional near-infrared spectroscopy (fNIRS). Against this backdrop, it is proposed that the areas of consumers' neural activations will differ according to not only the level of fashion involvement but also VM types, which leads to the final hypothesis:

### Hypothesis 3

Depending on the level of consumers' fashion involvement, the activated brain regions upon three VM types are different.

## Materials and methods

### Ethics statement

The experiment was conducted upon the approval of the University Institutional Review Board, Pusan National University. Written consent was obtained from all subjects prior to starting. The experimental procedure was conducted in accordance with the ethical standards described in the latest Declaration of Helsinki.

### Participants

A total of twenty healthy subjects (all women in her age 20~39, ten in their 20s, ten in their 30s) participated in this experiment. All of them had normal health status and had no brain-related history. Based on the fNIRS study (ex.『Three Class Classification of fNIRS Signals for the Detection of RGB Color Stimuli in the Visual Cortex』, 『Negative emotions impact lateral prefrontal cortex activation during theory of mind: An fNIRS study』), there were 8~15 general subjects, and 20 people were calculated by considering the validity of the existing measured data. Before the experiment, the procedure, operation, and experimental contents of the fNIRS system were fully explained, and the study was conducted only after obtaining informed written consent. The subjects were asked to remain still and relax while focusing on the visual stimulus shown to them.

### Visual stimuli

The stimuli used in this research were created using Adobe Photoshop CS 6 based on Kim and Lee [8], who proposed three different VM types to simulate a real storefront.

**Fig 1:**
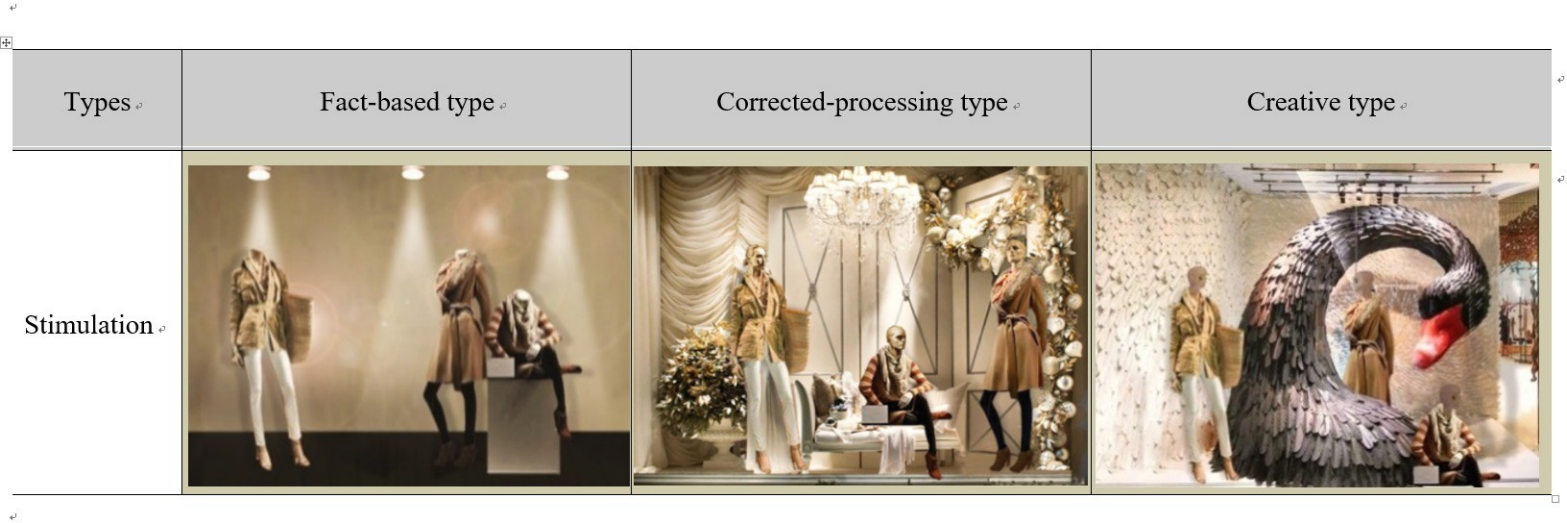
Stimuli by the fashion VM types.

Kim and Lee [8] analyzed 724 photos to define three VM types and derive ways of expression. Based on this, stimuli for each type of VM were simulated. To minimize visual effects other than fashion VM components, the products were presented in the same way, and the types and arrangements of mannequins, objects, and accessories were changed to create one stimulus for each of the three VM types. In addition, to check the manipulation of the stimuli, 21 fashion experts (Over 3 years of working for a fashion company) were asked to use an online Google questionnaire to identify significant differences in the stimuli for each type. The stimuli are shown in Fig 1.

### fNIRS

Portable and wearable neuro-imaging devices, including EEG and fNIRS, have greatly facilitated the emergence of neuroscience [61]. fNIRS is a newly developed optical imaging method to measure the hemodynamic responses in the cerebral cortex. This technology uses two wavelengths of near-infrared light to measure cerebral oxy-hemoglobin (HbO) and deoxy-hemoglobin (HbR), in contrast to the BOLD signal (HbR) of fMRI [62–65]. It is safe, handy, easy to use, and affordable [66–68]. Moreover, it can be worn while walking or dancing[69–71], and it is non-invasive. Given these attributes, fNIRS provides an attractive way to continuously monitor the brain dynamics of study participants, whether stationary or on the move. In this experiment, to capture brain signals, 16 channel probes were placed on the frontal cortex using a multi-channel fNIRS near-infrared spectroscopy imaging system (CW-fNIRS, DYNOT 232, sampling rate 1.81Hz, USA).

### Procedure

**Fig 2:**
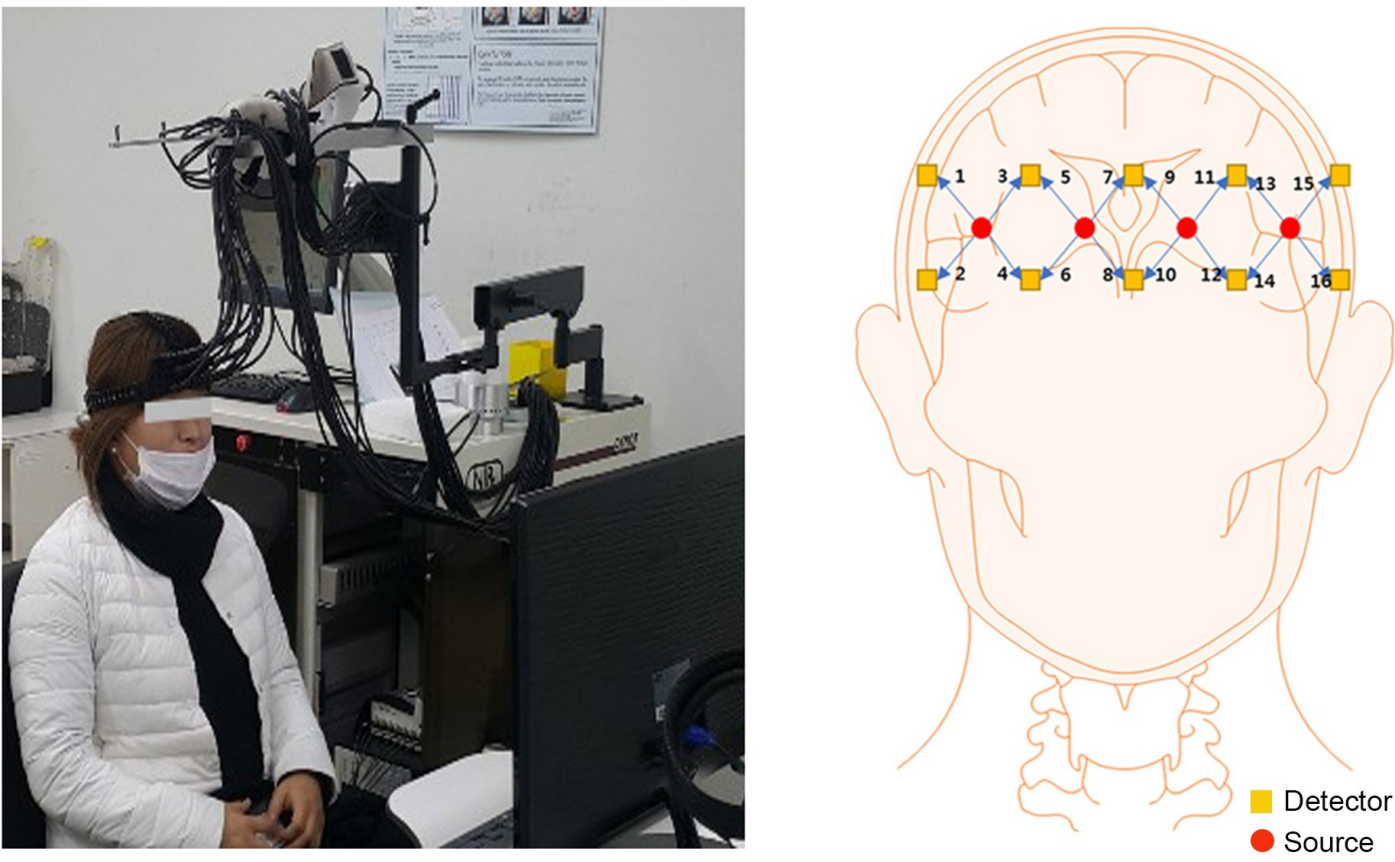
Experimental environment and the arrangement of optical sensors on the prefrontal cortex.

**Fig 3:**
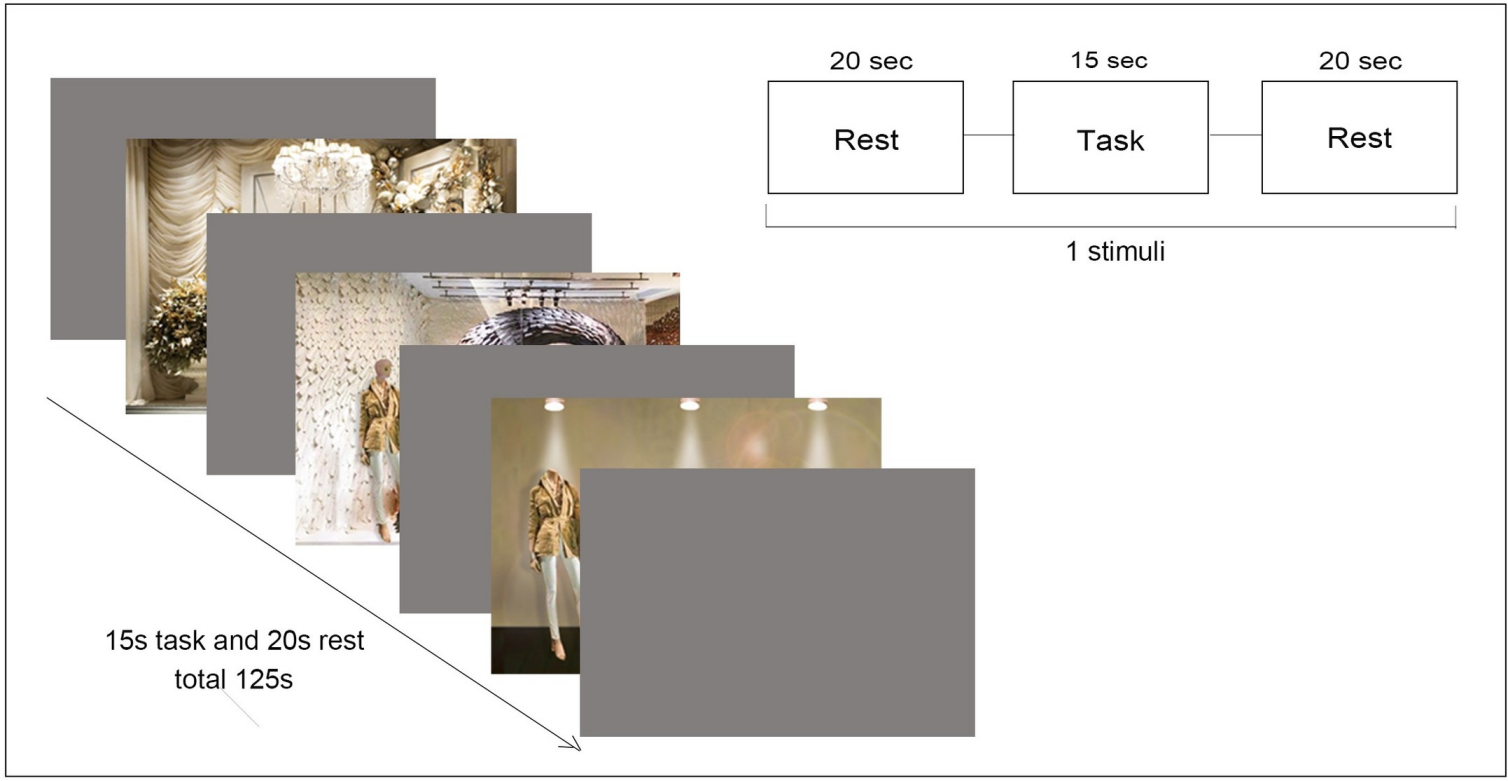
Experiment paradigm.

Before the experiment, a survey was conducted to gather information about demographics and the level of fashion involvement of the subjects. A total of 8 questions were asked to the subjects to measure their level of fashion involvement, based on the research of [72] and [40]. Visual stimuli were projected on a screen (Samsung LED model: LS24A300) in front of the subjects. The distance between the subject and the screen was about 50 cm, and the resolution was 1980 × 1380 pixels. A 20-second break was given between each 15-second stimulus. An experimental session consisted of 125 seconds, including the initial 20 sec calibration and 105 sec task, see Figs 2 & 3.

### fNIRS measurement and analysis

To analyze fNIRS data, NIRS_SPM, an open-source software programmed by MATLAB ®2015a (Math-works, Natick, MA) was used [73]. The light intensities measured with the fNIRS device were used to calculate concentration changes of HbO and HbR (i.e., *Δ*C_HbO_(*t*) and *Δ*C_HbR_(*t*)) using the modified Beer-Lambert law (MBLL) as follows.

$$\left[\begin{array}{c} \Delta C_{\mathrm{HbO}}(t)\\ \Delta C_{\mathrm{HbR}}(t) \end{array}\right]={\left[\begin{array}{cc} \alpha_{\mathrm{HbO}}(\lambda_{1}) & \alpha_{\mathrm{HbO}}(\lambda_{2})\\ \alpha_{\mathrm{HbR}}(\lambda_{1}) & \alpha_{\mathrm{HbR}}(\lambda_{2}) \end{array}\right]}^{-1}\left[\begin{array}{c} \Delta A\left(t,\lambda_{1}\right)\\ \Delta A\left(t,\lambda_{2}\right) \end{array}\right]\frac{1}{L\cdot DPF},$$ (1)

where *ΔA*(*t*, *λ*_*j*_) represents the variation in the absorbance of the near-infrared light emitted at wavelength *λ*_*j*_, *α*_HbX_(*λ*_*j*_) indicates the extinction coefficient of HbX in μMmm^-1^, *DPF* is the unitless differential path length factor, and *L* denotes the emitter-detector distance (mm). To remove the respiration and cardiac noises, a fourth-order Butterworth low-pass filter with a cutoff frequency of 0.15 Hz was used. Also, to eliminate the low-frequency drifting, de-trending was performed using the NIRS-SPM software.

Based on the modeled function and data for each channel, the statistical similarity was calculated using a *t*-test. The *t*-test was conducted using MATLAB’s “robustfit” function: It can be presumed that brain response occurred in the corresponding channel when the obtained *t*-value is over 1.6. The channels where brain responses were expected were marked as a region of interest (ROI). Averages by stimuli and subject groups were calculated to understand the characteristics of the graph (mean, peak, slope, skewness, and kurtosis values) and to find criteria of distinction according to the fashion VM types. Brain activation observed in the prefrontal cortex of the subjects according to different VM types was mapped using the obtained *t*-values.

## Results

### Brain activation of consumers by the VM types

**Fig 4:**
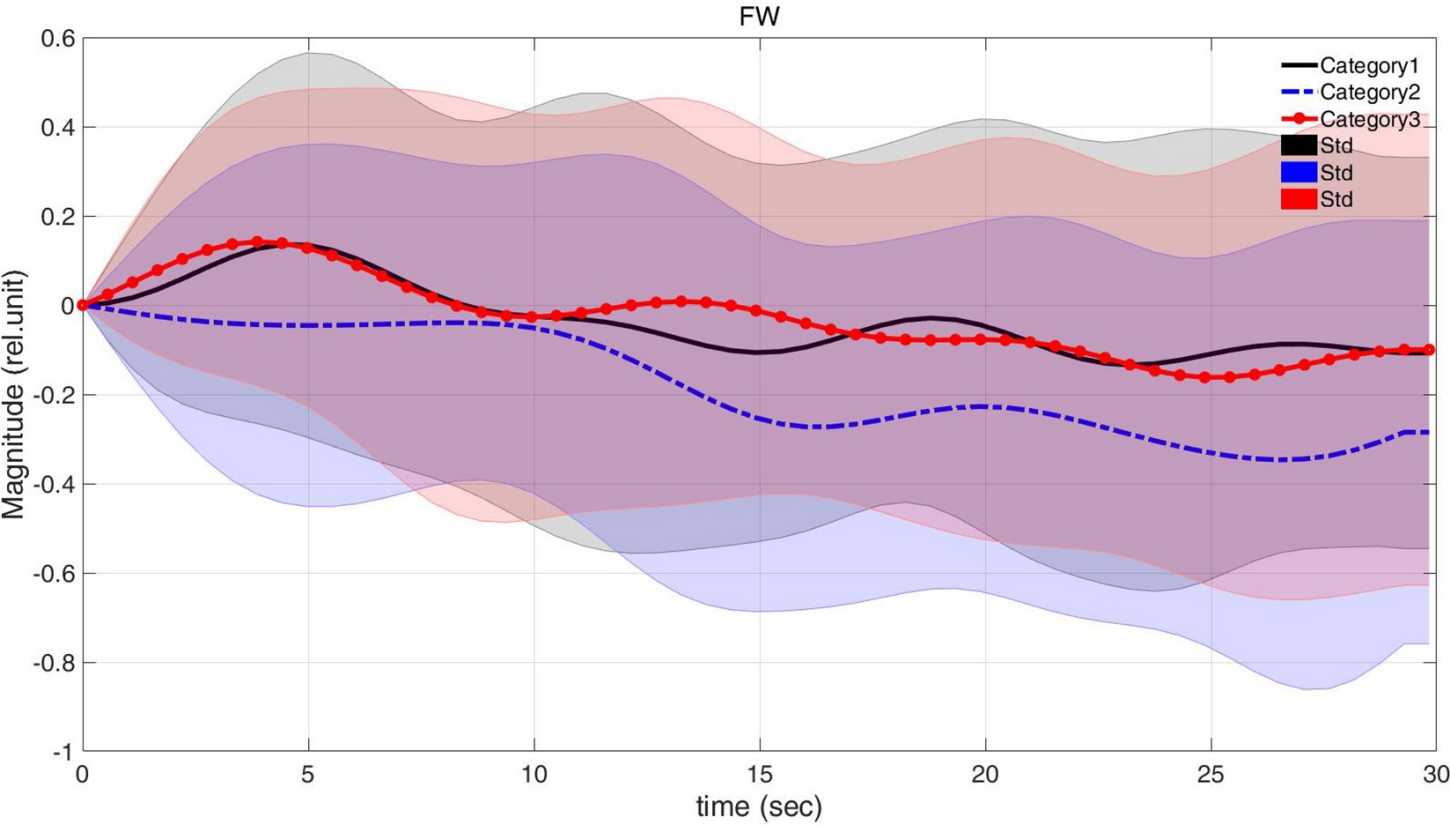
Comparison of the averaged HbOs according to VM types (Category 1: Fact-based, Category 2: Corrected-processing, and Category 3: Creative fashion VM).

To examine consumers’ brain activation according to three types, the average values of the ROI channels for all subjects based on individual categories (fact-based, corrected-processing, and creative) were compared in Fig 4. The non-parametric statistical analysis had been performed (Table 2). There were a significant difference of peak values between corrected-processing and creative fashion. Also, the peak values of creative fashion was the most high among the categories and the lowest in the corrected-processing.

**Table 2:** Non-parametric statistical analysis (Wilcoxon rank sum test) of peak value.

Category	Mean	std	Wilcoxon rank sum test	
			Category	*p*-value
Category 1	0.165	0.295	Category 1&2	0.365
Category 2	0.126	0.209	Category 2&3	0.046*
Category 3	0.398	0.213	Category 1&3	0.732

*: *p*-value < 0.05.

### Brain activation by the level of fashion involvement

**Fig 5:**
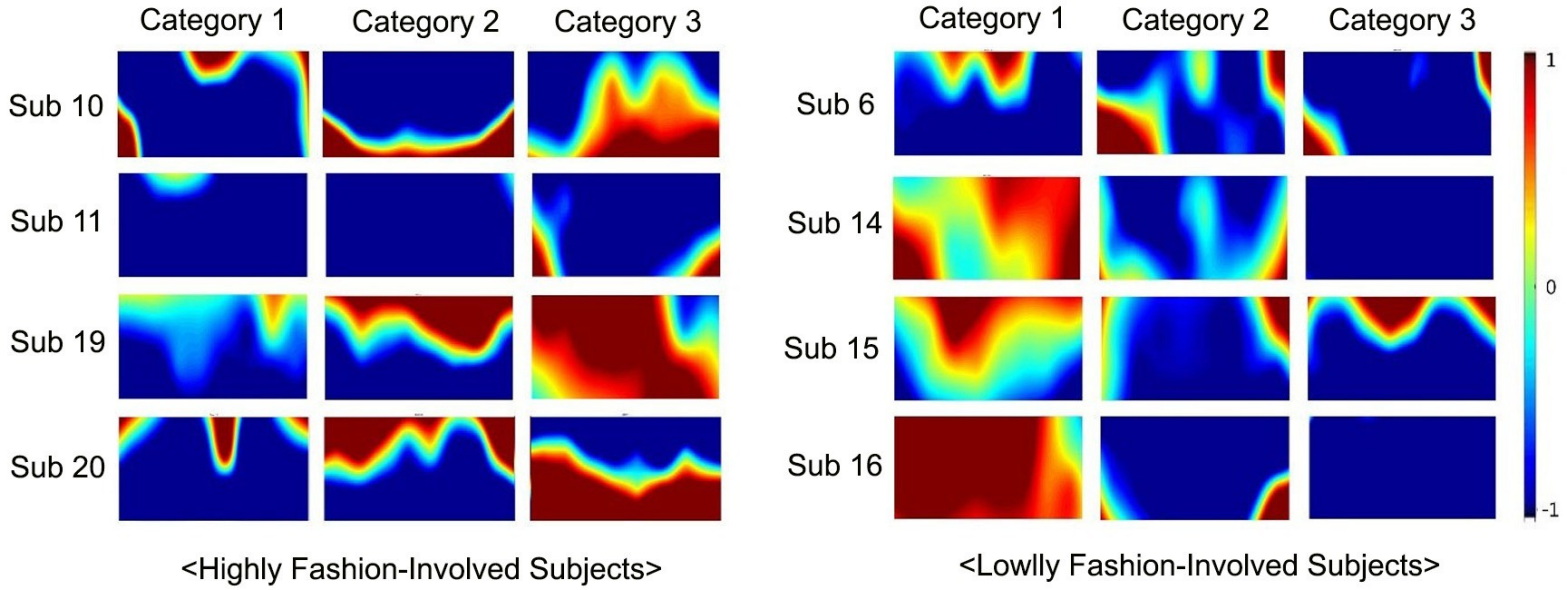
Comparison of brain activity maps between highly vs. lowly fashion-involved groups (four subjects in each group).

The brain activity map of four subjects representing the high-involvement group and four representing the low-involvement group were compared to observe differences in brain activation, see Fig 5. The results reveal that individual brain activations depend on their level of fashion involvement. Highly fashion-involved participants showed an active brain response to the creative fashions, while the brains of low-involved participants were activated more in response to fact-based styles [74].

### Brain activated channels for highly fashion-involved subjects

**Fig 6:**
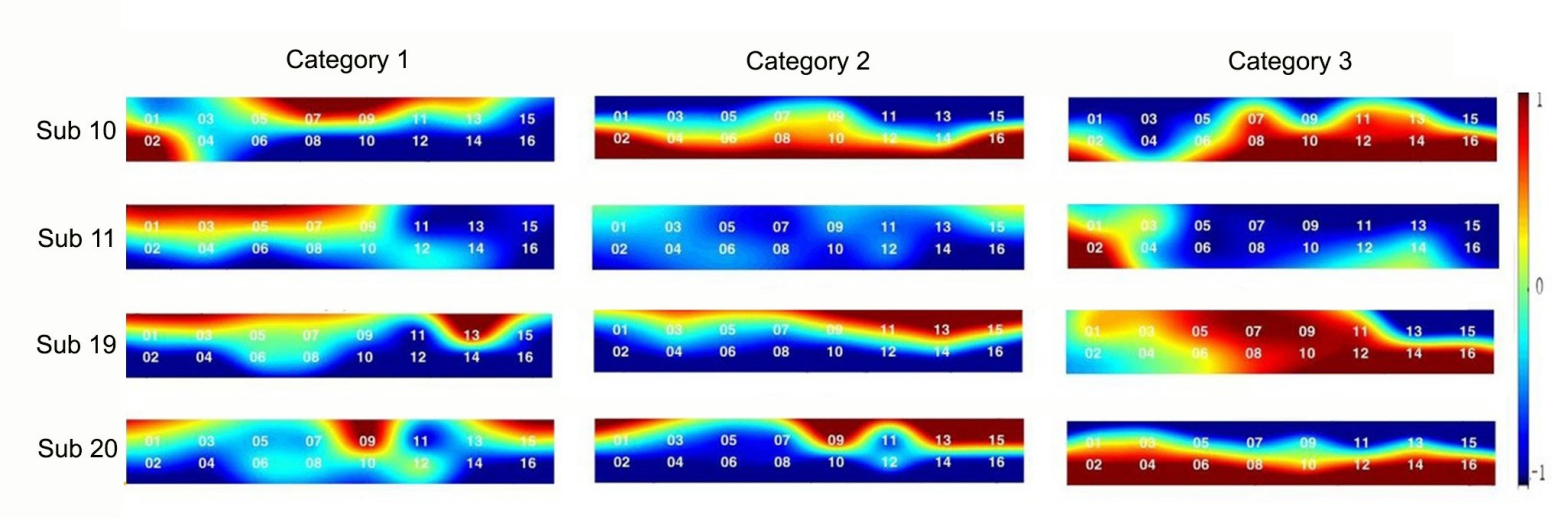
Brain activation maps of four subjects representing highly fashion-involved group.

**Fig 7:**
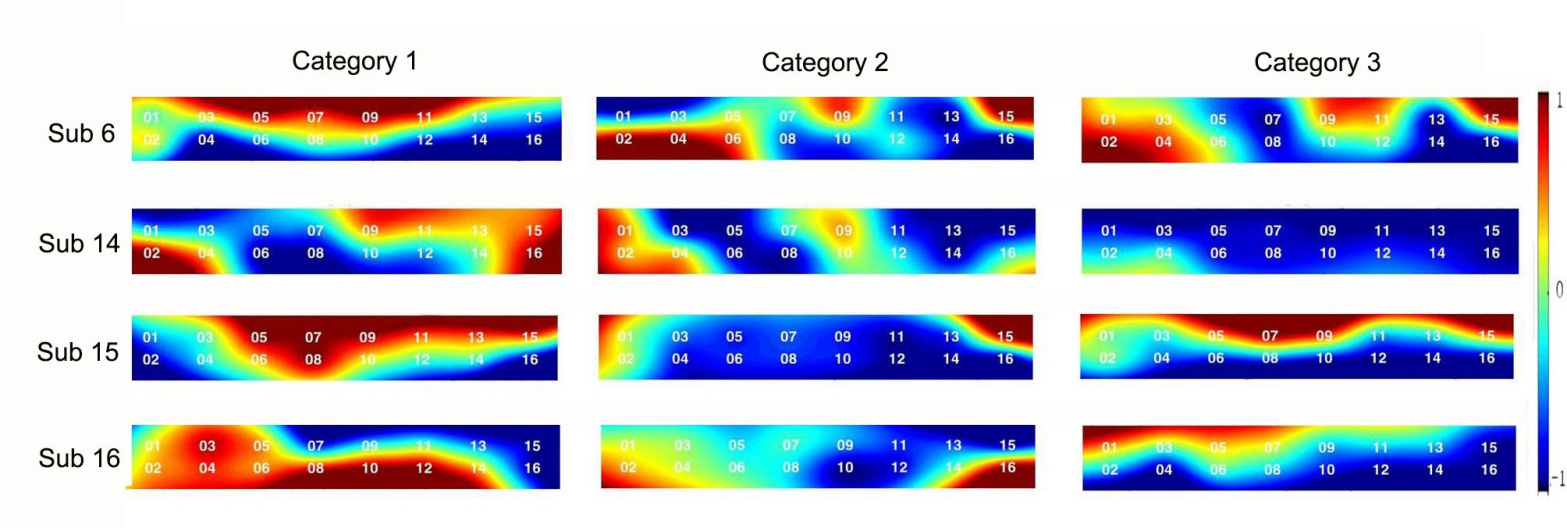
Brain activation maps of four subjects representing lowly fashion-involved group.

The difference in brain activation by channels was observed by dividing the subjects into two groups, including four subjects with low fashion involvement and four subjects with high fashion involvement. As a result, a review of the activation channels according to the level of fashion involvement for the three VM types showed significant differences only in the creative stimuli. The t-values, which indicate the degree of hemodynamic changes associated with the stimuli, were analyzed by two groups using Wilcoxon rank sum test. In Table 3, the results represented the activated brain region, which activated differently depending on the groups, i.e., channels 6, 8, 10, 12, 13, and 14, see Figs 6 and 7.

**Table 3:** Activated channels of 4 subjects representing highly fashion-involved group and low involved group.

Channel	Category 3 Creative type fashion VM				
	*p*-value	Mean (highly fashion-involved subjects)	std	Mean (lowly fashion-involved subjects)	std
ch1	0.382	-2.500	5.748	-5.268	5.383
ch2	0.645	-2.581	6.296	-3.447	6.163
ch3	0.235	-2.816	4.727	-5.434	5.208
ch4	0.130	-2.108	6.309	-6.268	7.087
ch5	0.382	-4.235	5.096	-5.839	4.755
ch6	0.038^*^	-2.718	4.053	-7.502	3.497
ch7	0.505	-3.368	5.028	-5.926	6.897
ch8	0.021^*^	-1.196	4.105	-8.151	4.400
ch9	0.279	-1.483	7.832	-6.571	7.462
ch10	0.010^*^	-0.448	5.946	-8.123	4.748
ch11	0.279	-1.958	7.603	-6.898	6.613
ch12	0.010^*^	0.337	7.745	-8.331	6.624
ch13	0.050^*^	-0.972	6.705	-8.810	6.828
ch14	0.015^*^	0.929	8.527	-8.384	7.217
ch15	0.382	-0.422	9.068	-6.137	7.230
ch16	0.235	-1.303	7.149	-7.211	6.507

*: *p*-value < 0.05.

## Discussion and conclusions

### Theoretical implications

The brain activation of the subjects showed significant differences according to the fashion VM types. When they were exposed to the creative VM, their response was notably higher. A difference was also observed in brain activation depending on the level of fashion involvement: Highly fashion-involved subjects showed higher brain activation to creative VM, while those with low-level of fashion involvement showed more brain activation for the fact-based VM (see Fig 5). Finally, activated channels shared by the subjects also differed depending on the VM types.

It is noteworthy that highly fashion-involved subjects had their OFC region activated by the creative VM, unlike the fact-based or corrected-processing types. Recently, researchers have discovered a wealth of evidence concerning the relation of the OFC with emotional empathy. It has been confirmed that the OFC is activated in response to emotional empathy rather than cognitive empathy, through experiments where subjects were asked to infer the thoughts or emotion of the protagonist in a [75] or of the characters in a short scenario [76]. Kida et al. [77] researched the areas of the activated brain by gentle touch of a hand, and found the OFC is especially related to the pleasant feeling created by touch. Based on these findings, it can be assumed that highly fashion-involved subjects had emotional compassion for the creative VM.

This research revealed that brain activation shows significant differences according to different types of fashion VM, and these responses are influenced by the level of fashion involvement of consumers. Therefore, corporations or brands will greatly benefit by applying the appropriate VM to match their consumers’ targets and goals. In particular, it is important to develop creative type VM to reach highly fashion-involved consumers so that these drivers and influencers can be mobilized and inspired. It is expected that this research will serve as a reference for future studies using neuroscience, and enable more objective and scientific fashion VMs to be created based on the characteristics of different consumers.

### Practical implications

In the complex modern society and under intensifying competition, a strategic fashion VM must be implemented to suit the purpose and situation for consumer critical burial operations. Since the results of this study show different brain responses to VM types depending on the level of fashion involvement, it will be useful for companies or brands to understand better the level of fashion involvement of consumers targeted and create a store with the appropriate VM type. It is especially important for consumers with high fashion involvement to develop and apply new methods season by season so that the brain region involved in emotional empathy can be activated through creative VMs.

### Limitations and future research

A potential limitation of this study is that the stimuli type is limited to the window of the fashion store. Future research should focus on different VMs in the fashion store for more subjects. In this study, we proposed a new methodology for future fashion VM research instead of using traditional surveys. Discovering how consumers’ brains react in a fashion retail environment and learning how to use such knowledge, companies and brands may be able to offer consumers more empathic solutions through fashion.

## Supporting information

S1 Dataset The data set of 20 subjects’ hemodynamic responses occurred by different visual merchandising types. For further understanding our study, recorded fNIRS data were provided as supporting information.
(ZIP)
