# Correction: Is consumer neural response to visual merchandising types different depending on their fashion involvement?

**DOI:** 10.1371/journal.pone.0253360

**Published:** 2021-06-10

**Authors:** The PLOS ONE Staff

The first author’s name is spelled incorrectly. The correct name is: Hyoung-Suk Kim. The publisher apologizes for the error.

The ORCID iD is missing for the first author. Please see the author’s ORCID iD here:

Author Hyoung-Suk Kim’s ORCID iD is: 0000-0003-2102-8785
